# Addressing Medical Deserts in Europe: Lessons From a Comparative Analysis

**DOI:** 10.1111/hex.70606

**Published:** 2026-03-09

**Authors:** Alicja Domagała, Katarzyna Dubas‐Jakóbczyk, Ana Isabel Gonzalez Gonzalez, Robert Likic, Kamila Michalska, Iwona Kowalska‐Bobko, Christoph Sowada, Linda Flinterman, Sorin Dan, Ronald Batenburg

**Affiliations:** ^1^ Institute of Public Health, Faculty of Health Sciences Jagiellonian University Medical College Krakow Poland; ^2^ Innovation and Research Promotion Department Research and Education General Directorate, Health Ministry Madrid Spain; ^3^ School of Medicine University of Zagreb Zagreb Croatia; ^4^ Netherlands Institute for Health Services Research (NIVEL) Utrecht the Netherlands; ^5^ School of Management University of Vaasa Vaasa Finland; ^6^ Department of Sociology Radboud University Nijmegen the Netherlands

**Keywords:** health workforce shortages, medical deserts, recruitment and retention of health workers, regional health policies

## Abstract

**Background:**

European countries face a common problem of medical deserts—areas where the population has limited access to healthcare services.

**Objective:**

This article aims to define medical deserts and provide an in‐depth overview of the factors driving desertification and the solutions applied to reduce its negative consequences across six European countries: the Netherlands, Spain, Poland, Croatia, Germany and Ireland.

**Methods:**

Applied methods include five consecutive steps: (1) development of a case study template; (2) defining criteria and selecting case study countries, (3) desk research, (4) consultation with national experts and (5) comparative analysis.

**Results:**

There is no formally recognised definition of medical deserts in all the analysed countries. Nevertheless, the concept is often associated with sparsely populated, isolated areas with limited access to healthcare services. The factors driving desertification are similar across the six countries and include: ageing and depopulation, health workforce deficits, unattractive working conditions and geographical factors. Solutions implemented to mitigate the negative effects of medical deserts can be classified into several broad categories: health workforce planning and monitoring, training and career pathways, innovative healthcare models, support mechanisms and infrastructure development.

**Conclusions:**

The factors driving medical deserts in European countries are complex and multidimensional. In consequence, the policy approaches aimed at limiting their negative consequences also require a comprehensive approach. Addressing medical deserts requires focusing on both the supply and demand sides of health services provision and comprehensive strategies tailored to each country's or region's specific circumstances.

**Patient or Public Contribution:**

This study was conducted within the ROUTE‐HWF project framework, which emphasised the importance of patient and public involvement throughout the research process. Their contribution was particularly valuable during national and international workshops with key stakeholders, including community representatives, healthcare professionals and policymakers. These stakeholder workshops focused on discussing the root causes of medical deserts and exploring national and regional strategies to address them. This inclusive approach ensured that the analysis of the factors driving desertification and potential solutions incorporated the perspectives and lived experiences of affected populations.

**Abbreviations**
EU: European Union
GDP: gross domestic product
GP: general practitioner
HWF: health workforce
MoH: Ministry of Health
NUTS: Nomenclature of Territorial Units for Statistics
OECD: Organisation for Economic Co‐operation and Development
PHC: primary healthcare
PPS: purchasing power standard

## 1 Background

The issue of ‘medical deserts’ is becoming an increasingly significant challenge across Europe, threatening equitable access to healthcare services and the sustainability of health systems [1]. Although the term 'medical desert’ is not universally accepted and its application varies considerably across countries, it consistently refers to areas where individuals experience insufficient access to the health workforce (HWF) and services [2]. Most of the existing research on medical deserts has focused on non‐European countries, including Australia, New Zealand, the United States and Canada [2]. In the United States, the term ‘underserviced’ has been used for several decades to identify communities or populations with limited access to medical care, especially primary care. This has led to the formal designation of 'health professional shortage areas’ and 'medically underserved areas’ [3]. In the European context, the term ‘medical deserts’ has been used for the last decade (initially in France [4]) to describe areas where the population has insufficient access to healthcare services, resulting in unmet healthcare needs, either partially or entirely, due to the lack of access and/or improper service quality [5, 6].

The need for explicit policies and practices to support HWF in medical deserts has increased significantly since the COVID‐19 pandemic [7]. The situation is particularly challenging in rural areas and economically disadvantaged urban regions, which face specific difficulties in attracting and retaining healthcare professionals [3, 8, 9]. Medical deserts pose substantial barriers to access to healthcare services [2] and can negatively affect population health [9]. Evidence suggests that poor health outcomes are associated with longer patient travel time: those who live farther from a healthcare facility tend to have worse health outcomes (e.g., survival rates, length of hospital stay) [8]. Moreover, delays or avoidance in accessing healthcare services contribute to increased morbidity and mortality related to both acute and chronic illnesses, including mental health disorders [2].

The lack of a precise definition and categorisation of medical deserts poses a challenge to policymaking, hindering their ability to develop a comprehensive understanding of this phenomenon. This can lead to confusion in research and policy discourse, resulting in misleading comparisons and recommendations [5]. While European countries recognise that medical deserts are a significant problem and are implementing measures to address them, the lack of specific policy rationales or targets makes it difficult to define—and therefore select—the most appropriate policies [2, 6, 9].

The study objectives were to (1) identify how medical deserts are interpreted in selected European countries (the Netherlands, Spain, Croatia, Poland, Germany and Ireland), (2) provide an in‐depth overview of the factors contributing to medical deserts and (3) identify the solutions/approaches implemented to mitigate the negative consequences of medical deserts. This work was conducted as a part of the European Union project: *A Roadmap out of medical deserts into supportive health workforce initiatives and policies* (ROUTE‐HWF) [10].

The added value of this study lies in its examination of how medical deserts are conceptualised across European countries, offering a comparative perspective on different health systems. By analysing this concept in diverse national contexts, the study provides a more comprehensive understanding of its complexity and policy relevance.

## 2 Methods

We used a qualitative comparative methodology based on a case study approach. This research method involves a mixture of data collection techniques and a systematic, grounded theory development process to analyse the existing phenomena [11]. It is useful in analysing novel research dilemmas to address multidimensional and complex theoretical gaps [11]. Specifically, we used the Walt and Gilson Policy Triangle Framework [12] to structure our analysis of the context, actors, processes and policy content associated with the causes of medical deserts and the solutions implemented to address them. The research followed the ROUTE‐HWF project's work plan and involved five consecutive stages: (1) development of a case study template; (2) defining criteria and selecting case study countries; (3) conducting desk research; (4) consulting national experts; and (5) conducting comparative analysis.

To guide cross‐country interpretation, we used an approach informed by health systems and governance theories [13]. These theoretical frameworks ensured coherence between the study objectives and strengthened the interpretative validity of our comparative analysis.

### 2.1 Development of a Case Study Template

A case study template was developed based on a previously conducted scoping literature review on medical deserts characteristics [2] as well as insights generated during national and international key stakeholder workshops. As part of the wider ROUTE‐HWF project activities, these workshops included community representatives and members of the public, who provided insights into the drivers and effects of medical deserts. Their contributions informed the analytical framework developed for the case studies and supported the interpretation of contextual factors across countries.

The template covered three main sections: (1) general country contexts, including sociodemographic, health system and HWF characteristics; (2) types/definitions of medical deserts and factors driving desertification; and (3) national policies and approaches to mitigate medical deserts [14].

### 2.2 Defining Criteria and Selecting Case Study Countries

Five main criteria were established to guide the selection of the case study countries:

1. Geographical diversity (e.g., country size, mainland or island status).
2. Population characteristics (e.g., total population, population density).
3. Economic resources (e.g., health expenditure as a percentage of GDP, health spending per capita).
4. HWF characteristics (e.g., number of practising medical doctors and nurses, number of medical graduates).
5. Data availability (e.g., availability of the national experts and comprehensive data from previous ROUTE‐HWF project activities, including key stakeholders' workshops).

The selection process was designed to ensure heterogeneity across countries, capturing a broad spectrum of national, geographical and organisational contexts. This allowed for a more comprehensive, pan‐European comparative analysis. This selection approach followed the principles of comparative case study design, ensuring the internal and external validity of the findings. Based on these criteria, the ROUTE‐HWF project's expert panel selected the following six countries: the Netherlands, Spain, Poland, Croatia, Germany and Ireland. The main characteristics of these countries are presented in Table 1.

**Table 1:** Characteristics of the six countries included in the analysis.

Category	Indicator/feature	The Netherlands	Spain	Croatia	Poland	Germany	Ireland
Type of healthcare system organisation	Social health insurance (SHI) vs. tax‐based national service (NS)	SHI administered by competing health insurers	NS with decentralised national coordination	SHI with centralised public payer	SHI with centralised public payer	SHI with a multipayer system	NS with centralised governance
Geographical characteristics	Area (thousands km^2^)	41.9	506	56.6	312.7	357.1	70.3
	Share of area on island (%)	0.0%	1.0%	5.6%	0.0%	0.0%	100%
Population characteristics	Total population (million)	17.94	48.62	3.86	36.62	83.46	5.35
	Population 65+ or over (%)	20.5%	20.4%	23.0%	20.5%	22.4%	15.5%
	Population density in a country (per 1 km^2^)	526.0	96.2	69.0	119.4	235.8	77.4
	Rural population (% of total population)	4%	20%	42%	40%	18%	36%
	Range of population density per region (per 1 km^2^)—from the region (NUTS 2) with the lowest to the highest ratio	188.4–1079.2	25.5–6124.2	43.6–1274.1	54.1–544.7	69.9–4311.6	37.6–182.9
Economic characteristics (health expenditures)	Current health expenditures as % of gross domestic product (GDP)	10	9.2	7.1	8.1	12.3	6.9
	Current health expenditures, purchasing power standard (PPS) per inhabitant	4 847.70	3 136.73	2 032.13	2 240.16	5 413.31	4 473.47
HWF characteristics	Practising medical doctors per 100.000 inhabitants	391.5	438.9	401.4	385.6	466.3	379.1
	Share of medical doctors aged 55–64	16.5%	24.9%	22.8%	20.9%	23.0%	14.7%
	Range of the number of practising medical doctors per 100.000 inhabitants—from the region (NUTS 2) with the lowest to the highest ratio	125.29– 612.55	340.55–601.57	n/a	215.12–733.59	375.98–640.27	n/a
	Medical doctors graduates per 100.000 inhabitants	13.97	13.62	17.06	15.83	12.23	25.04
	Practising nurses and midwives per 100.000 inhabitants	1136.59	616.44	291.1	666.75	1258.3	1445.79
	Nurses graduates per 100.000 inhabitants	63.48	23.98	44.86	20.03	43.69	32.01

*Note.* Eurostat data for 2023 or the latest available; ‘n/a’ —data not available.

### 2.3 Desk Research

Comprehensive desk research of published data sources was conducted for each case study country (information in both English and the national languages was involved). The data sources included research papers, grey literature, national strategy statements, legal and policy regulations and thematically related reports developed by the OECD, the WHO European Observatory on Health Systems and Policies, the European Commission and European projects dedicated to HWF. Additionally, a quantitative analysis of data from national statistical offices and/or Eurostat Statistics was performed. For each country, the following indicators were analysed over time and (if available) per region: population density, health expenditures and HWF availability per type of profession (both practising and graduates). The data collected for each country were synthesised using the standardised case study template described in Section 2.1.

### 2.4 National Expert Consultations

The draft versions of the case study materials were validated and updated in consultation with national experts. Experts were recruited through the networks of the ROUTE‐HWF consortium partner institutions; however, they were external specialists and not affiliated with the participating institutions. The experts were selected through purposive and snowball sampling based solely on their recognised experience and knowledge of policies related to HWF, health system organisation and medical deserts. Experts were approached via email or telephone and invited to participate in the consultation process. Follow‐up communication took place via virtual meetings, telephone, or written correspondence, depending on the availability of the experts. A minimum of two experts (representing research/academia and health policy) were consulted for each country. Fourteen experts participated in the study in total—three from both Spain and Poland, and two from each of the four remaining countries. The experts had diverse professional backgrounds, including public health (2), HWF planning (4), healthcare providers (4) and health system management (4). Half of the experts were women, with an average of 15 years’ professional experience.

They were asked to validate and, if needed, update the preliminary case study findings. Box 1 in the Supporting file presents the consultation guide used across countries. Based on information provided by experts, the case study materials were expanded and refined. Each country study report was developed through an iterative process, involving successive rounds of feedback and additional discussions or email correspondence to clarify any discrepancies. All inputs were cross‐checked and triangulated with secondary data sources (e.g., national databases and European sources) to ensure consistency and accuracy across countries.

### 2.5 Comparative Analysis

To explore the similarities and differences in defining medical deserts, as well as mapping their contributing factors and solutions across the six included countries, a comparative qualitative analysis was performed. Information from all six case studies was analysed, and the results were grouped and synthesised. Deductive thematic analysis with manual coding was carried out, and the results were categorised according to the three core elements of the medical deserts characteristics: (1) the interpretation of medical deserts in each country, (2) the factors driving desertification and (3) the solutions applied to mitigate the negative effects of medical deserts.

To ensure analytical neutrality, the comparative analysis was verified by a subgroup of three researchers, who were independent from the authors of the six case studies. The synthesis was based mainly on the national case study reports, supplemented by expert input and secondary data sources (e.g., EU, WHO data). To ensure data triangulation and reliability, the study findings were cross‐verified across sources and iteratively discussed within the research team until consensus was reached.

## 3 Results

Table 1 presents the main indicators of the six analysed countries’ geographical, population, economic and HWF characteristics. The data show significant differences among the chosen countries. For example, the population density ranges from 69 inhabitants per 1 km² in Croatia to as much as 526 in the Netherlands. The percentage of GDP allocated to healthcare ranges from around 6,9% in Ireland to more than 12% in Germany. The number of practising medical professionals per 100,000 inhabitants ranges for medical doctors from 379 in Ireland to 466 in Germany, and for nurses and midwives from only 291 in Croatia to 1446 in Ireland.

### 3.1 Interpretation and Definition of Medical Deserts in the Analysed Countries

**Figure 1:**
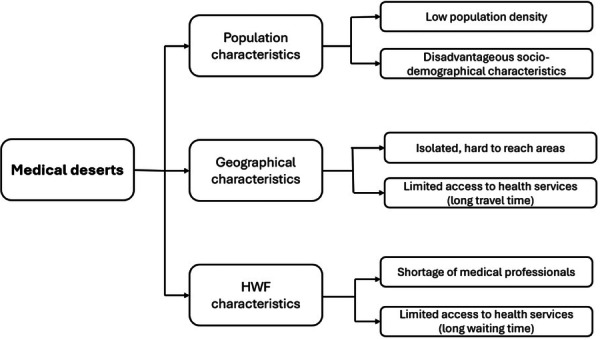
Conceptual framework of key elements underpinning ‘medical deserts’.

In all analysed countries, there is no formal, commonly used, or recognised definition of medical deserts. The terms often used in formal national documents/reports usually relate to the specific characteristics applicable to the region (e.g., ‘isolated area’), characteristics of the population (e.g., ‘depopulating area’), or limited access to care or resources (e.g., ‘undersupplied’, ‘underserved area’). In all countries, medical deserts are associated with areas characterised by low medical care supply and high demand. They are often located in the peripheral areas, which have lower population density and corresponding reduced access to healthcare services (Table 2). In summary, the term medical desert is most often associated with specific characteristics of population, geographical area and HWF (Figure 1).

**Table 2:** Interpretation of the term ‘medical deserts’ in the analysed countries.

Country	The Netherlands	Spain	Croatia	Poland	Germany	Ireland
Definitions	no legal definition most precise administrative terms: areas with strongest population decline (depending on the ratio: shrinking/depopulating (‘*krimpgebied*’); potentially depopulating (‘*anticipeergebied*’) labour market shortages (‘*personeelstekort*’)	no legal or administrative definition popular term ‘Empty Spain’ (*España Vaciada)* refers to rural provinces in the interior with progressing depopulation	lack of formal definition; the term is indirectly used to refer to areas with hard‐to‐reach healthcare or areas of special state concern	no legal definition white spots *(białe plamy)* or areas with significant staff shortages	lack of formal definition underserved areas *unterversorgte Gebiete*	no formal definition *‘red zones’* ‐ counties with a supply of a health service 10% lower than the national average
Examples of medical deserts	areas with shortages of GPs (*huisartsentekorten* mostly rural and depopulating regions along the country's border Provinces of Zeeland and Fryslân	mostly rural areas with restricted access to healthcare Region Aragon; smaller Canary Islands; western zones of Madrid Region	significant differences between urban vs. rural areas, and between northern and southern Croatia Slavonia, the Dalmatian coast, and the Croatian islands	‘white spots’ located all over the country (municipalities without a PHC doctor) the regions with serious staff shortages: Lubuskie, Opolskie, Warmińsko‐Mazurskie	mostly rural, remote, sparsely populated areas with restricted access to healthcare workers in the ‘new federal states’, e.g., Brandenburg, Mecklenburg‐Western Pomerania, Saxony‐Anhalt, Thuringia and Saxony	mostly rural, depopulating areas on the east coast of Ireland e.g., the Kildare, Meath, Wexford, and Wicklow counties

*Note*. Based on the ROUTE‐HWF project. Deliverable 7.1. Case study report [14].

The most precise terms are found in the Netherlands and were formally introduced by the government: shrinking or depopulating area (‘*krimpgebied*’), potentially depopulating area (‘*anticipeergebied*’), or labour market shortages area (areas with ‘*personeelstekort*’). These areas are classified as those experiencing the most significant population decline, with shrinking areas projected to lose 12.5% of their population by 2040, and anticipatory areas expected to decline by 2.5% [14]. In the Netherlands, regulatory norms are set by the government. For example, the maximum travel time to a hospital is set at 30 min, and to a general practitioner (GP) practice at 15 min. The GP population ratio is relatively low in rural and depopulating regions along the country borders, and forecasts for the coming years indicate a growing GP shortage.

In Spain, the term ‘medical desert’ (*desiertos medicos*) is not used in legal, administrative, or policy documents. It is usually associated with sparsely populated and isolated areas, which are considered key characteristics. Some depopulated autonomous communities in Spain are referred to as ‘*empty Spain’*. Other relevant factors include, most notably, geographical accessibility, distance to nearest health facility and access to health workers (Table 2).

In Croatia, medical deserts are characterised as areas with hard‐to‐reach healthcare, and the term is indirectly referenced through the designation of *special state concern areas*. Significant differences are observed between urban and rural areas, as well as between northern and southern Croatia. Examples of medical deserts identified in our research include Slavonia, the Dalmatian coast and the Croatian islands.

In Poland, the term used is ‘*white spots’ (białe plamy)* or areas with significant staff shortages defined as areas lacking service (due to medical staff deficit or long waiting time). These ‘*white spots’* can be found all over the country.

In the German context, the term ‘medical deserts’ (*unterversorgte Gebiete)* is associated with sparsely populated areas and limited access to healthcare workers. Statistical data on the number and distribution of doctors show that there is no national shortage of medical doctors, but recruiting doctors (especially GPs) for regions with the highest proportion of rural areas is a challenging task.

Also in Ireland, the term ‘medical deserts’ is not commonly used or recognised. In 2019, the report ‘Geographic profile of healthcare needs and non‐acute healthcare supply in Ireland’ [15] indicated significant inequalities in the supply of primary, community and long‐term care across Irish counties. Counties with a supply of a health service 10% lower than the national average are defined as ‘red zones’ with the most difficult situation.

### 3.2 Factors Driving Desertification

Numerous drivers contribute to the development of medical deserts in the analysed countries. They were grouped into four main categories: geographical factors, population characteristics, HWF characteristics and work‐related factors (Table 3). It can be seen that, depending on the country, the intensity and scope of particular types of desertification factors vary. In the next section, we elaborate on the four types of desertification factors.

**Table 3:** Factors driving desertification in six analysed countries.

Factors	The Netherlands	Spain	Croatia	Poland	Germany	Ireland
Geographical factors	In rural areas, there is a greater distance to basic facilities (including GPs, schools, sports centres).	Mountainous, isolated areas Rural areas have the worst access to basic local services	Insular nature and mountainous territory	Long distance to medical facilities and poorly developed public transport network	Limited number of medical facilities and other infrastructure	Lack of adequate infrastructure in rural areas
Population characteristics	Ageing Depopulation Changing in population needs	Ageing Depopulation Changing in health needs of the population	Ageing Depopulation Significant disparities in the demand depending on seasons (summer vs. winter)	Ageing (including HWF) Depopulation Increase in healthcare needs	Ageing Changing the health needs of the population	Population growth Increasing demand for healthcare services
Health workforce characteristics	Unequal distribution of medical staff (rural vs urban areas)	Unequal distribution of HWF across communities Increased emigration and decreased immigration	Uneven distribution of health professionals Shortages of HWF Migration of young doctors	Significant staff shortages and uneven distribution Poor HFW planning mechanism Migration of HWF	Uneven distribution Shortages of medical staff Migration of HWF	Understaffing Uneven distribution of doctors Migration of HWF
Work‐related factors (in rural, remote, isolated areas)	Lack of job opportunities in economically underserved and depopulated areas	Heavy workload Inflexible work/employment regulations Difficult working conditions Long distances travelled by rural doctors	Lack of comprehensive regulatory frameworks Inadequate scope of practice Unclear responsibilities	Long working time Heavy workload Administrative burden Limited career prospects Unsatisfactory remuneration	Heavy workload Inadequate use of resources Difficult working conditions	Unattractive working conditions Long working hours Unsatisfactory work‐life balance Lack of proper financial incentives

*Note*. Based on the ROUTE‐HWF project. Deliverable 7.1. Case study report [14].

#### 3.2.1 Geographical Factors

Geographical factors strongly influence the existence and persistence of medical deserts in Spain and Croatia, where mountainous and isolated areas, as well as islands, contribute to unequal distributions of medical infrastructure and doctor density. In Spain, numerous rural regions face significant challenges accessing basic local services, including health clinics, schools, hospitals and sports centres [16]. Insufficient economic and communication infrastructure (including Wi‐Fi) further hinders access to medical care. In Croatia, regions and islands that are not popular tourist destinations face the challenges of extreme weather conditions, poor internet connectivity, a lack of public transportation and limited hospital facilities. In the case of islands, transportation difficulties—such as sea crossings and limited ferry services, especially during the off‐season—contribute to longer travel times. In all six analysed countries, unequal access to healthcare services between urban and rural areas remains a key challenge, with access to public transport playing a pivotal role.

#### 3.2.2 Population Characteristics

Population characteristics reveal common trends and challenges in the analysed countries, affecting the provision of healthcare services and contributing to the appearance of medical deserts. The share of the population aged 65+ has increased significantly across all countries, and further growth is expected [17]. Population ageing and depopulation impact both healthcare needs and the location of healthcare resources and are key factors that influence desertification. The migration of young people to bigger cities for better economic opportunities leaves an older population with increased healthcare needs in depopulating areas. In all the analysed countries, the population is unevenly distributed across regions, with more densely populated urban areas and sparsely populated rural/remote regions. Specifically, Croatia experiences significant disparities in the demand for medical assistance, especially in regions with fluctuating populations, that is, on the Adriatic coast during the summer season [14]. There is a significant influx of people, but a much smaller population remains during the winter. The seasonal variation leads to the emergence of medical deserts, where the demand for HWF drastically exceeds the off‐season capacity [14].

#### 3.2.3 HWF Characteristics

The unequal distribution of medical staff between rural and urban areas appears to be a common issue in all six countries. In Spain, while there is no overall shortage of HWF, an uneven distribution exists among autonomous communities [18]. Similar disparities are observed in Germany, where there is also no national physician deficit but where the HWF concentration in urban/academic centres is high, leaving rural regions underserved [19]. In Poland, a substantial shortage of doctors and nurses is a major challenge for the health system, with a deficit of specialists indicated by the MoH since 2012 [14].

In Ireland, the recruitment and retention of HWF in rural/remote areas is challenging due to reported limitations in career advancement, inadequate support networks and a lower perceived quality of life [14]. Croatia faces similar issues exacerbated by social and economic challenges, with underdeveloped regions experiencing significant HWF shortages, partly driven by emigration (mainly young medical doctors). In 2020, Croatia was among the three EU countries with the largest number of emigrating doctors [14]. The impact of emigration is also evident in Spain, Poland and Ireland, caused by better professional opportunities and higher standards of living abroad; problems with work‐life imbalance; long working hours; unsatisfactory salaries; and heavy workloads [20, 21]. This is particularly evident among young professionals, who place greater value on work‐life balance and are less willing to work long hours. HWF ageing is also a major concern, especially in Poland, Germany and Spain. The share of healthcare workers older than 60 is increasing steadily, and when a GP in some rural/remote areas retires, there are no candidates for replacement [14]. Overall, the analysed countries face common HWF challenges, including distribution disparities, emigration trends and the need for targeted interventions to address shortages and improve working conditions.

#### 3.2.4 Work‐Related Factors

Difficult working conditions and limited career prospects in economically underserved and depopulated areas strongly contribute to medical deserts. The demanding working conditions in remote/rural areas (e.g., long working time, long patient lists, solitude and a lack of peer support [21]) drive the location preferences of young health workers, who often prefer specialisation with higher salary prospects as well as work in bigger cities with modern infrastructure and better employment opportunities [2, 22]. In all analysed countries, healthcare professionals seem less willing to practice in areas with poor infrastructure (e.g., schools, sports centres) and limited employment opportunities for their partners. In medical deserts, health workers often have to travel long distances to reach patients, especially in rural areas where the population is dispersed [14]. Among rural Spanish doctors, 45% travel more than 50 km/day to see patients and up to 5% travel more than 200 km [23]. Working in medical deserts can also be isolating, as health workers may not have access to the same level of professional development opportunities or collaborative networks as colleagues in more urban areas [14].

### 3.3 Approaches/Solutions Implemented to Reduce the Negative Effects of Medical Deserts

A variety of implemented measures/actions to strengthen HWF in underserved areas and improve access to healthcare services have been identified across the analysed countries. They were classified into five broad categories: planning and monitoring HWF distribution; training (undergraduate and postgraduate) and career pathways; innovative healthcare models; support and infrastructure; and other solutions (Table 4).

**Table 4:** Approaches/policies implemented to reduce the negative effects of medical deserts in six countries.

Approach	The Netherlands	Spain	Croatia	Poland	Germany	Ireland
Planning and monitoring HWF distribution	*Stock and flow model* to estimate the required number of GPs in training *Care and Welfare* Prognosis Model	*Human Resources Commission* of Interterritorial Council of National Health System	*Atlas of Medicine;* comprehensive overview of medical doctors’ distribution	*Maps of health needs* (geographical distribution of different health professionals)	*SICHERSTELLUNGSATLAS‐* interactive map developed by the National Association of SHI Physicians	*National Strategic Framework for Health & Social Care Workforce Planning* *Geographic profile of healthcare needs & nonacute healthcare supply*
Training (undergraduate and postgraduate) and career pathways	Promoting careers in medical professions to improve inflow and attract young people into healthcare education	Increase in the number of postgraduate training places for doctors; Local training for GPs Connecting nursing and medical schools with rural areas Erasmus Rural – summer practices for final‐year students	Increase in the number of undergraduate places for doctors	Significant increase in the number of undergraduate places and postgraduate training places for doctors Higher remuneration for doctors in residency training in family medicine Loan programmes for medical students	*German Concept for the Longitudinal Integration of rural medical training content and Experience in Medical Studies* (MilaMed project) *Longitudinal Curriculum in General Medicine to Strengthen Family Medicine Care in Rural Regions* (LOCALHERO)	Increasing the number of medical training places and expanding postgraduate training programmes in underserved areas
Innovative models of care	Task‐shifting E‐health solution (including patient portals; home monitoring for patients) Home healthcare models Nurse specialist Care coordination	Telemonitoring programme for primary care patients Proactive monitoring of chronically ill patients in complex situations, supported by innovative IT solutions Advanced nursing practice model	Provision of medical services by a ‘rotating *doctor*’ between 3 and 4 villages Skill‐mix/task‐shifting Significant role of nurse practices in areas without a doctor	Coordinated care New professionals (care coordinators) Skill‐mix solutions IT solutions	Strengthening coordinated care and practice Physician assistants as support for doctors Community health nurse Skill‐mix Mobile clinics (patients buses)	Telemedicine and e‐health initiatives Multi‐disciplinary teams Implementation of six new *Regional Health Areas*
Support mechanisms and infrastructure development	Local language training support for GPs Local housing support for GPs Innovative transport solutions (e.g., self‐driving, free‐of‐charge shuttle buses)	Offering housing for GPs willing to work in rural/isolated areas Investing in PHC infrastructure Attracting doctors from other regions by offering higher salaries	Tax exemption for doctors under 30 years of age. Favourable mortgages for young doctors Additional salaries and accommodation are covered by local authority Emergency helicopters for faster transport	Scholarships offered by local authorities Additional remuneration for doctors working in rural areas	Financial incentives to GPs who establish their practices in rural areas Subsidies to cover investment costs when taking over an existing practice or setting up a new one, or to finance fee supplements, training allowances, and scholarships	Rural practice support, including financial incentives and student loans
Other solutions	Cross‐border care Community involvement, including volunteers	Developing cooperation with the social care sector and emphasising local community involvement		Simplification of regulations on employment of health professionals from Ukraine	Integration of migrant health professionals	*The Non‐EU Rural GP programme* (with substantial educational support and clinical supervision)

*Note.* Based on the ROUTE‐HWF project. Deliverable 7.1. Case study report [14].

#### 3.3.1 Planning and Monitoring HWF Distribution

The Netherlands, Germany and Ireland have a longstanding tradition of planning and forecasting HWF. The Dutch use a stock‐and‐flow model to estimate the required number of GPs to train at the national level, advising ministries and educational institutions on maintaining a balance between HWF supply and demand [24]. In 2017, Ireland issued the National Strategic Framework ‘Working Together for Health’ to develop integrated projections for the Irish health and social care workforce [25]. To ensure the proper distribution of doctors in Germany, several measures have been taken, for example, ‘2020 Medical Studies Master Plan’ [26], which was agreed upon by the Federal Government and the states, allows the reservation of up to 10% of all study places for applicants who commit to working as GP in underserved/or at‐risk regions [27].

In Poland, Spain and Croatia, ROUTE‐HWF research has deemed HWF planning insufficient and neglected for many years [14]. More recently, interactive online tools/platforms have been developed, e.g., the Polish *Maps of Health Needs* (with data on the geographic distribution of HWF) [28] or the Croatian *The Atlas of Medicine* [29], with a comprehensive overview of healthcare providers.

#### 3.3.2 Training (Undergraduate and Postgraduate) and Career Pathways

In most countries, activities aimed at increasing the number of training places for doctors have been implemented (at both undergraduate and/or postgraduate levels). Also, policies aimed at training and recruiting young GPs in underserved/rural regions are commonly adopted. In the Netherlands, a central placement system for GP residents has been implemented to fill all training spots and encourage medical students to stay in their study region, indirectly aiding in GP distribution nationwide [14]. In Germany, the Joint Admissions Office allocates medical school places, reserving some for candidates committed to practising in areas of special public need or settling as GPs in underserved regions. The German Master Plan includes funding for the ‘RegioMed’ project, promoting medical studies in small towns and rural areas through a national virtual information platform [26]. The Master Plan also promotes general medicine as a way to ensure universal access to GPs [14]. Eleven out of sixteen German federal states offer financial incentives to GPs who establish practices in rural areas [30]. A notable example is the postgraduate training programme in Baden‐Württemberg, which aims to enhance access to primary healthcare (PHC) by increasing the appeal of general medicine [31]. To attract medical students to practice in rural areas, the German MoH has allocated more than 23 million euros to dedicated initiatives, including the ‘MiLaMed’ [32], ‘MEDIC’ [33] and ‘LOCALHERO’ [34] projects, which promote family medicine in rural regions.

The Polish MoH has attempted to reduce or eliminate shortages in ‘deficit specialisations’, including family medicine, by providing higher salaries during postgraduate training. This initiative has increased family medicine training for doctors by nearly 133% since 2015 [14]. Other initiatives include local governments’ scholarships for medical students, where recipients usually take up work in a particular county for a defined period [14]. In Spain, the General Council of Official Colleges of Physicians recommends that rural centres offer medical residency training to facilitate generational change [23]. There are also examples of regional initiatives in which summer practices for students are organised in rural areas (‘Rural Erasmus’), encouraging them to familiarise themselves with these regions and consider them for future employment [35].

#### 3.3.3 Innovative Models of Care

##### 3.3.3.1 Skill Mix/Task Shifting

Task shifting/skill‐mix modifications to broaden the scope of practice in rural/underserved areas (including medical deserts) are often used in the analysed countries to reduce the negative effects of staff shortages. Task‐shifting experiences in the Netherlands show its potential in addressing HWF deficits, improving care quality and optimising the right skill‐mix [36]. In Germany and Croatia, skill‐mix policies extend nurses’ competencies/responsibilities to include tasks traditionally performed by doctors [14]. Similarly, in Poland, nurses and midwives are authorised to issue prescriptions and medical orders, paramedics can provide medical emergency services and healthcare assistance, and physiotherapists are empowered to conduct independent physiotherapeutic visits [37]. Spain has also implemented broader skill‐mix policies, especially in PHC, by establishing multidisciplinary teams to address the limitations of solo practices and provide more comprehensive services for patients with chronic conditions and multimorbidity. This has been accompanied by task shifting to other professionals, mainly nurses and healthcare assistants. For example, in rural areas of the Navarra region, an advanced nursing practice model was implemented within multidisciplinary teams of administrative staff, nursing professionals, social workers, doctors, psychologists and physiotherapists [38]. In all countries, the challenges of implementing task‐shifting are similar, including legal barriers, resistance from professionals losing tasks and insufficient training budgets [14, 36].

##### 3.3.3.2 Models of Managing Complex Health Conditions

Coordinated care models are also used. They are usually defined as ‘a core function of team‐based primary and community care that delivers systematic, responsive and supportive care to people with complex chronic disease care needs’ [39]. In Valencia, a new model to coordinate care between PHC and hospitals for patients with complex needs was implemented and later expanded to other communities. The model introduced new nursing roles: hospital and community care managers, both equipped with dedicated tools, including guidelines and IT solutions. In Poland, the coordinated ‘PHC Plus’ model was implemented, and the competencies of doctors and nurses were expanded to comprehensively manage certain chronic conditions [37]. Evaluation of both the Polish and Spanish experiences found extensive benefits for patients and healthcare facilities [38, 40].

##### 3.3.3.3 E‐Health Solutions

Digital tools are widely used in healthcare, and the COVID‐19 pandemic has greatly accelerated their adoption in all the analysed countries. E‐prescriptions and e‐referrals are broadly used, but there are also online applications and dedicated portals/websites for patients. The use of e‐health tools increased from 42% in 2019% to 79% in 2021 in Dutch GP practices [41]. These tools can address challenges of the ageing population and staff shortages in underserved areas, for example, by enabling home monitoring [41]. An innovative application, the ‘Traffic light’, was initially piloted in Breda in the Netherlands (before being nationally implemented) to enhance care coordination and support patient transitions from hospital to district nursing in real‐time [14]. Another example is the ‘*Dolce Vita’* project, a data‐driven system facilitating acute elderly care redistribution to facilities through collaboration among nursing homes, GPs and hospitals [42].

In Spain, successful e‐health solutions include the Galician Health Service's telemonitoring programme for PHC patients [43]. The system enables remote health monitoring with colour‐coded indicators for targeted interventions [14, 43]. Patients can input health data via smartphones, and the system supports secure communication between patients and healthcare providers. This project was recognised by European CIRCE‐Join Action: Transfer of Best Practices in Primary Care as a best practice in PHC and was proposed for implementation in other countries [44]. In Andalusia, an innovative IT‐supported monitoring project effectively manages complex situations for chronically ill patients, using an online platform for telephone follow‐ups, in‐person visits and comprehensive assessments. Patients use a mobile telephone app for advice, recommendations and vital sign monitoring, enabling automated data collection [14]. Ireland's 2021–2023 Sláintecare also recommends technology‐driven remote healthcare services, which are especially beneficial for those in rural/remote areas [45].

##### 3.3.3.4 Healthcare at Home

Various innovative home care programmes, which often employ elements of e‐health solutions, have also been implemented to address the impact of medical deserts in the analysed countries. For example, a project in Tilburg introduced nurse specialist ambulance services featuring collaboration between local PHC and hospitals [46]. Home healthcare projects often involve collaboration between social services and local civic organisations (focused on supporting independent living) and medical care services.

#### 3.3.4 Support Mechanisms and Infrastructure Development

Staff shortages in all six countries are particularly significant in smaller communities or regions, especially in rural areas. To attract candidates to 'unattractive‘ areas, some local authorities in Poland and Croatia provide salary supplements or offer accommodation for recruited doctors [14]. In Poland, doctors who had passed the State Medical Examination were allowed to practice PHC in rural/low population density areas without completing a specialisation. Dutch local initiatives include additional language training for doctors starting jobs in Fryslân and assistance with housing and job searches for spouses in Zeeland. In Zeeland, there is the ‘vacation doctor’ programme, wherein GPs can work during vacation periods with free accommodation for their families, facilitating familiarity with the local environment [14]. The Croatian county government in Bjelovar‐Bilogora offers young doctors favourable mortgage/credit lines and supports the needs of younger families in the county [14]. There are also innovative transport solutions for patients (e.g., free‐of‐charge shuttle buses between the hospital and bus stops in the Netherlands or emergency helicopters in Croatia) [14].

#### 3.3.5 Other Solutions

Other solutions involve cross‐border care (e.g., between Dutch provinces and Belgian hospitals, as well as diverse community involvement projects and migrant workers initiatives. Community involvement is essential in healthcare collaboration, particularly in low‐populated/underserved areas, for citizen‐centred and sustainable healthcare systems [47]. In the Netherlands, both volunteers and informal care providers play significant roles, and the nation benefits from a robust tradition of volunteering [48]. Similarly, in Spain, enhancing cooperation with the social care sector and emphasising local community involvement has been shown to reduce the demand for PHC services.

In Germany, immigrant health workers play a crucial role in addressing staff deficits [49], and in the last decade, the proportion of active foreign‐trained medical doctors in the country has increased from 6.6% to 13.8% [50]. The Irish ‘Non‐EU Rural GP Programme’ aims to address GP shortages in rural areas by allowing GPs from outside the EU to work in routine rural GP practice for 2 years, with ongoing educational support and clinical supervision provided by the Irish GP College and the hosting GP practice [51].

## 4 Discussion

Our study has revealed that across the six analysed countries, there is no formal definition of ‘medical deserts’, and different characteristics can be used to classify given areas as medical deserts. The following terms are associated with medical deserts: *sparsely populated, isolated areas* (Spain), *underserved* or *undersupplied areas* (Germany, Ireland), *white spots* (Poland), *shrinking areas/depopulating areas* (the Netherlands), or *areas with hard‐to‐reach health care* (Croatia).

Despite differences in the exact terminology across countries, the factors contributing to medical deserts and the solutions applied to mitigate them are often similar and can be classified into a few clear categories. Nevertheless, the scope and intensity of a given dimension and/or a particular factor/solution can differ between countries and regionally within a given country.

Our results are in line with the findings of other European research projects focused on medical deserts: AHEAD [52], and OASIS [53], as well as the evidence reported in international literature [2, 3, 4, 5, 8, 9].

The causes of medical deserts are multifaceted, including geographical, population‐related, HWF‐related and work‐related issues. Working conditions of HWF in many countries are often described as difficult, characterised by understaffing, long working hours, a fast working pace and unsatisfactory work‐life balance, with many workers experiencing burnout [1, 22]. Healthcare professionals employed in underserved areas often face a heavier workload, as well as having to travel long distances to reach patients [14]. The multidimensional character of the factors contributing to medical deserts necessitates a comprehensive set of approaches to mitigate their negative consequences.

Our results indicate that the analysed countries are following international recommendations in this area [1, 6] by applying multidimensional policies for both healthcare supply and demand sites.

The health policy responses identified in our study can be summarised as the five main levers for tackling medical deserts. Firstly, planning and monitoring measures aim to improve the HWF distribution through stronger data systems and forecasting tools. Secondly, actions relating to training and career pathways aim to attract and retain HWF in underserved regions. Thirdly, innovative healthcare models (e.g., skill mix, IT solutions) help to maintain access to healthcare in areas facing serious staff shortages. Fourthly, support and infrastructure interventions (e.g., housing support) make work in remote areas more feasible. Finally, context‐specific measures emphasise the importance of cross‐border care, community involvement, collaboration with the social sector and the integration of non‐EU and migrant healthcare professionals via simplified procedures.

Our approach and analysis are intended to support public authorities and health professionals in better understanding and exploring the factors driving desertification and tailoring potential solutions to mitigate/reduce the consequences of medical deserts. To eliminate medical deserts, it is essential to clearly understand their characteristics, considering that countries and regions vary in many aspects, including geographical characteristics, population characteristics and the density of HWF. While most solutions/approaches were developed as systemic/national solutions, regional/local initiatives were also present. These activities focus on attracting medical doctors to rural areas, offering flexible work arrangements for better work‐life balance, promoting rural lifestyles and supporting family arrangements. Many innovative home healthcare programmes (often incorporating e‐health solutions) have also been introduced to reduce the consequences of medical deserts. As presented in our study, in many countries, efforts are being made to encourage independent living for older individuals; however, the growing number of single‐person households results in vulnerable groups dependent on local care and support [2, 9].

An important initiative is the involvement of communities in closer cooperation with healthcare workers. Community engagement has been recognised as a key factor in developing citizen‐centred and sustainable healthcare systems, particularly in low‐populated or underserved areas [47]. In the Netherlands, both volunteers and informal care providers play a significant role in the social and healthcare systems. The country has a strong tradition, and consequently a high level of volunteering compared with other European nations [48]. Dedicated online platforms connect volunteers with available opportunities across the country or region, matching them based on interests, competencies and availability. This approach is especially relevant given the increasing proportion of elderly patients, many of whom live alone and experience loneliness and a lack of social support. It would be beneficial for such initiatives to be adopted and promoted in other European countries as well.

### 4.1 Limitations

Our study is not free of limitations. First, the purposive sampling of national experts results in the risk of selection and information bias. This limitation was mitigated by referring to the published literature whenever available. Moreover, our six case‐study countries presented a range of circumstances but may not represent the full range of conditions across all European countries. Another limitation of the study is the inconsistent and limited comparability of available statistical data on the HWF across countries and their regions.

### 4.2 Implications for Future Research

Our analysis suggests several areas for future research, including investigating the impact of specific solutions on healthcare access and population health outcomes. It also provides guidance for policy initiatives, particularly in fostering multi‐stakeholder collaboration and developing intersectoral approaches. Finally, it is worth noting that limited access to health services is not only related to geographical areas, but also to social inequalities affecting minority, migrant and other vulnerable groups. Such disparities can lead to feelings of neglect and loss of trust in public health policies and institutions. A more systematic exploration of these issues, particularly concerning users’ experiences and perceptions, should be a priority for future research.

## 5 Conclusions

Our analysis addresses a significant gap in the existing research by exploring definitions of medical deserts, their contributing factors and the solutions implemented to mitigate them across six European countries. We provide evidence that the factors driving desertification are multifaceted, including geographical, population‐related, HWF‐related and work‐related aspects. The intensity and relevance of these factors are country‐specific. Our study indicates that the complex nature of medical deserts requires the implementation of multidimensional and intersectoral approaches.

## Author Contributions

**Alicja Domagała:** conceptualization, data curation, formal analysis, investigation, methodology, supervision, validation, writing – original draft, writing – review and editing **Katarzyna Dubas‐Jakóbczyk:** conceptualization, data curation, formal analysis, investigation, methodology, validation, writing – original draft, writing – review and editing **Ana Isabel Gonzalez Gonzalez:** conceptualization, data curation, investigation, methodology, validation, writing – review and editing **Robert Likić:** data curation, investigation, methodology, validation, writing – review and editing **Kamila Michalska:** data curation, investigation, writing – review and editing **Iwona Kowalska‐Bobko:** data curation, investigation, validation, writing – review and editing **Christoph Sowada:** data curation, investigation, validation, writing – review and editing **Linda Flinterman:** conceptualization, data curation, investigation, methodology, validation, writing – review and editing **Sorin Dan:** conceptualization, methodology, project administration, validation, writing – review and editing **Ronald Batenburg:** conceptualization, data curation, formal analysis, funding acquisition, investigation, methodology, supervision, validation, writing – review and editing.

## Ethics Statement

The authors have nothing to report.

## Conflicts of Interest

The authors declare no conflicts of interest.

## Supporting information


**Box 1:** The ROUTE‐HWF project's ‘Case studies on medical deserts’ consultation guide.

## Data Availability

The data supporting the findings of this study may be available from the corresponding author upon reasonable request.
