# Supplementary material for: Addressing Medical Deserts in Europe: Lessons From a Comparative Analysis
Source: Health Expect. 2026 Mar 9;29(2):e70606. doi: 10.1111/hex.70606 (PMC12970483; doi:10.1111/hex.70606)
Supplement: Supplementary file 1 — Box 1: The ROUTE‐HWF project's ‘Case studies on medical deserts’ consultation guide. [file HEX-29-e70606-s001.docx]

**SUPPLEMENTARY FILE**

**Box 1. The ROUTE-HWF project's ‘Case studies on medical deserts’ consultation guide**

1. *Can you provide general comments on the draft version of the case study on medical deserts in your country?*

*For example, are there any additional sources of information not included in our draft document that are important for understanding the issue of medical deserts in your country?*

1. *Can you comment on the applicability of the Project’s approach to defining and measuring medical deserts?*
2. *Can you provide feedback on the list of factors contributing to medical deserts and the solutions to mitigate them described in the draft document?*

- *Are there any other factors, not mentioned in our draft document, that are relevant to your country or region and contribute to the creation and persistence of medical deserts?*
- *Can you comment on the list of policies we identified from the literature (and other sources) that have been implemented to mitigate or eliminate medical deserts in your country? In your opinion, how effective are these policies?*
- *Can you help us in mapping the factors contributing to medical deserts and the corresponding solutions, based on the specific type of medical deserts identified?*

1. *Are there any other approaches or policies implemented to address medical deserts in your country or region? What factors contribute to the success of policies aimed at mitigating medical deserts?*
